# Morquio‐B disease: Clinical and genetic characteristics of a distinct *GLB1*‐related dysostosis multiplex

**DOI:** 10.1002/jmd2.12065

**Published:** 2019-11-28

**Authors:** Iman S. Abumansour, Nataliya Yuskiv, Eduard Paschke, Sylvia Stockler‐Ipsiroglu

**Affiliations:** ^1^ Division of Biochemical Genetics BC Children's Hospital Vancouver British Columbia Canada; ^2^ Department of Pediatrics University of British Columbia Vancouver British Columbia Canada; ^3^ Department of Medical Genetics, Faculty of Medicine Umm Al‐qura University Makkah Saudi Arabia; ^4^ Department of Pediatrics Medical University of Graz Graz Austria; ^5^ BC‐Children's Hospital Research Institute Vancouver British Columbia Canada

**Keywords:** dwarfism, literature review, mucopolysaccharidosis, natural history, spondyloepiphyseal dysplasia

## Abstract

**Background:**

Morquio‐B disease (MBD) is a distinct *GLB1*‐related dysostosis multiplex involving the trabecular parts of long bones and spine, presenting a mild phenocopy of *GALNS*‐related Morquio‐A disease.

**Methods:**

We analyzed 63 (n = 62 published) cases with MBD to describe their clinical, biochemical and genetic features.

**Results:**

Forty‐one of 51 cases with informative clinical data had *pure MBD* including progressive growth impairment, kyphoscoliosis, coxa/genua valga, joint laxity, platyspondyly, odontoid hypoplasia. Ten of 51 had *MBD plus* neuronopathic manifestations including intellectual/developmental/speech delay, spasticity, ataxia dystonia. Corneal clouding, cardiac valve pathology, hepatosplenomegaly, spinal cord compression were infrequent and atlantooccipital dislocation, cardiomyopathy and cherry red spot were never reported. Urinary glycosaminoglycan and oligosaccharide excretion was consistently abnormal. Keratan sulphate‐derived oligosaccharides were only detected using LC‐MS/MS‐based methods. Residual β‐galactosidase activities measured against synthetic substrates were 0%‐17%.

Among 28 *GLB1* variants, W273 L (34/94 alleles) and T500A (11/94 alleles) occurred most frequently. W273L was invariably associated with *pure MBD*. *Pure MBD* also was reported in a case homozygous for R201H, and in the majority of cases carrying the T500A variant. Homozygous Y333C and G438E were associated with *MBD plus* neuronopathic manifestations. T82M, R201H, and H281Y, observed in seven alleles, previously have been found sensitive to experimental chaperones.

**Conclusion:**

Data provide a basis for future systematic collection of clinical, biochemical, morphologic, and genetic data of this ultra‐rare condition.

**Synopsis**

*GLB1‐*related Morquio‐B disease (MBD) is a distinct dysostosis multiplex resembling mild forms of *GALNS*‐related Morquio‐A disease and occurs as pure skeletal MBD and as *MBD plus* neuronopathic phenotype. The presence of at least one W273L allele determines *pure MBD*. Chaperone sensitivity has been shown in a variety of alleles associated with MBD.

## 1 BACKGROUND

*GLB1‐*related disorders are caused by a deficiency of β‐galactosidase, a lysosomal enzyme facilitating the degradation of complex carbohydrates bound to a variety of structurally unrelated molecules such as gangliosides, proteoglycans, and N‐ and O‐linked glycoproteins. The spectrum of clinical phenotypes is wide: Type 1 (infantile) GM1‐gangliosidosis (OMIM 230500) begins before age 1 year with hepatosplenomegaly, progressive loss of neurodevelopmental abilities and vision, cherry red macula spot, seizures, and dystonia/spasticity. Type 2 (late infantile/juvenile) GM1‐gangliosidosis is characterized by a later onset of motor and cognitive regression. Type 3 (adult) GM1‐gangliosidosis causes extrapyramidal signs, cardiomyopathy, and variable degrees of intellectual disability.^1^

*GLB1*‐related disorders are also associated with skeletal deformities (eg, kyphoscoliosis and short stature), which, like in other lysosomal storage diseases, nosologically have been classified as dysostosis multiplex.^2^, ^3^ Morquio‐B disease (MBD) (OMIM 253010)^4^ is a distinct form of *GLB1*‐related disorder presenting with a specific type of dysostosis multiplex which has been known as Morquio syndrome since its first description by *Morquio* 5 and *Brailsford*.^6^

Morquio syndrome is characterized by short stature with a disproportionally short trunk, kyphoscoliosis, pigeon chest (pectus carinatum), short neck, large appearing head with midface hypoplasia and mandibular protrusion, large appearing joints (elbows, wrists, knees, ankles), coxa and genua valga and flat feet. Joint laxity, corneal clouding, and cardiac valve disease and tracheal stenosis are additional findings. Characteristic radiological findings include platyspondyly and vertebral beaking, odontoid hypoplasia, spinal canal narrowing, hip dysplasia, dysplasia of the carpal and tarsal bones, as well as shortening and epi‐and metaphyseal dysplasia of long bones (eg, shortening of the ulna and sloping of the distal ends of radius and ulna).

Currently two genetic conditions are known to cause Morquio syndrome: *GALNS*‐related Morquio‐A disease (OMIM 25300) and *GLB1*‐related Morquio‐B disease (MBD). Keratan sulfate is a proteoglycan that accumulates in both Morquio‐A and Morquio‐B disease.

The *GLB1* gene contains 16 exons spanning more than 60 kb. The longest transcript variant (NM_000404.2) is a 2.5 kb mRNA giving rise to a 70 kDa precursor protein which is processed within the lysosomes into the 64‐kD mature β‐galactosidase enzyme protein.^4^, ^7^ *GLB1* alternatively gives rise to a 2.0 kb mRNA transcript, formed by splicing out exons 3, 4, and 6^8^ which encodes the elastin binding protein, a key‐recycling chaperone in the tropoelastin assembly process for elastogenesis in the extracellular matrix.^9^ The β‐galactosidase monomer consists of two β*‐*domains and a TIM barrel domain, which together generate appropriate protein folding and functional integrity.^4^, ^7^, ^10^ Mutations associated with Type 1/infantile onset GM1‐gangliosidosis, for the most part, are located in the core protein region causing β‐galactosidase instability, whereas mutations associated with milder phenotypes, such as types 2 and 3 GM1‐gangliosidosis, tend to be on the protein surface.^7^

Patterns and distribution of the accumulating substrates across the various tissues and organs are determined by the impact of the underlying *GLB* mutation on the molecular pathophysiology of the β‐galactosidase protein.^4^, ^11^ While accumulation of GM1‐gangliosides in the brain seems most responsible for neurologic manifestations in GM1‐gangliosidosis, excretion of both skeletal and corneal forms of keratan sulfate has been shown in MBD and type 1 (infantile) GM1‐gangliosidosis.^10^

More than 150 pathogenic *GLB1* variants are known with the vast majority being associated with GM1 gangliosidosis,^1^ whereas the number of variants described in association with MBD is rather limited. W273L is the most frequent MBD allele.^10^, ^11^, ^12^, ^13^, ^14^ Numerous other alleles have been found in both GM1‐gangliosidosis and in MBD.^11^, ^13^, ^15^, ^16^, ^17^ but the type and degree of overlap between pure skeletal and neuronopathic phenotypes is hard to predict.

We performed a literature review to revisit all cases previously published as *GLB1*‐related MBD, with the aims (a) to describe the clinical phenotype associated with MBD; (b) to compare the clinical data of all genotypes identified in this review against the classical W237L Morquio‐B allele. Additionally, we reviewed published data on potentials of small molecules for allele specific rescue of β‐galactosidase activity for those alleles identified in the reported MBD cases.

## 2 METHODS AND RESULTS

Our literature review enrolled English language PubMed‐listed publications and reports of cases diagnosed with MBD from its first description in 1976^18^ to June 2018. Search terms included: “GM1‐gangliosidosis,” “Morquio‐B,” “Mucopolysaccharidosis,” “Beta‐galactosidase deficiency,” “β‐galactosidase deficiency,” and “GLB1 deficiency.” In addition, we added data from three patients presented at the 13th International Symposium on Mucopolysaccharidoses and Related Diseases, Sauipe, Bahia, Brazil, August 13‐17, 2014^19^ and one thus far unpublished patient with MBD previously diagnosed at our center.

We identified 23 articles/reports containing information about 62 MBD cases. Including our own case (P1), 63 cases (22 male; 18 female; 23 gender not reported) met the inclusion criteria for our analysis: (a) clinical findings consistent with a Morquio phenotype; (b) diagnosis confirmed by demonstration of deficient β‐galactosidase activity and/or a homozygous/compound heterozygous *GLB1* mutation. We grouped all these cases according to availability of clinical information including skeletal and neuronopathic phenotype, as well as of *GLB1* variant data (Table 1).

**Table 1:** Summary of 63 cases, including n = 62 reported as *GLB1*‐related Morquio‐B disease and one unreported patient (case vignette 1)

Group	Cases	References
**Cases with informative clinical data (skeletal and neuronopathic)** • Cases with skeletal features only (*pure MBD*) • Cases with skeletal and neurologic/developmental features (*MBD plus)*	51 41/51 10/51	Arbisser et al 1977^20^; Bagshaw et al 2002^21^; Beck et al 1987^22^; Di Cesare et al 2012^23^; Giugliani et al 1987^15^; Groebe et al 1980^24^; Gucev et al 2008^25^; Hofer et al 2009^11^; Holzgreve et al 1987^26^; Ishii et al 1995^27^; Maroteaux et al 1982^28^; Mayer et al 2009^16^; O'Brien et al 1976^18^; Paschke et al 2001^13^; Paschke et al 2014^19^; Roze et al 2005^17^; Santamaria et al 2006^14^; Sheth et al 2002^29^; Sohn et al 2012^30^; Trojak et al 1980^31^; van Gemund et al 1983^32^; P1 (unpublished).
**Cases without informative clinical data** (data on skeletal or neuronopathic or both phenotypes missing)	12	Hinek et al 2000^33^; Oshima et al 1991^34^; Pronicka et al 1981^35^; Santamaria et al 2006^14^
**Cases with unknown genotype**	16	Arbisser et al 1977^20^; Beck et al 1987^22^; Di Cesare et al 2012^23^; Groebe et al 1980^24^; Holzgreve et al 1987^26^; Maroteaux et al 1982^28^; O'Brien et al 1976^18^; Pronicka et al 1981^35^; Sheth et al 2002^29^; Trojak et al 1980^31^; van Gemund et al 1983^32^
**Cases with reported genotype**	47	Bagshaw et al 2002^21^; Giugliani et al 1987^15^; Gucev et al 2008^25^; Hinek et al 2000^33^; Hofer et al 2009^11^; Ishii et al 1995^27^; Mayer et al 2009^16^; Oshima et al 1991^34^; Paschke et al 2001^13^; Paschke et al 2014^19^; Roze et al 2005^17^; Santamaria et al 2006^14^; Sohn et al 2012^30^; Case vignette 1 (unpublished)
**Cases with reported genotype and informative clinical data**	38	Bagshaw et al 2002^21^; Giugliani et al 1987^15^; Gucev et al 2008^25^; Hofer et al 2009^11^; Ishii et al 1995^27^; Mayer et al 2009^16^; Paschke et al 2001^13^; Paschke et al 2014^19^; Roze et al 2005^17^; Santamaria et al 2006^14^ (case MB2); Sohn et al 2012^61^

Fifty‐one of 63 reported cases contained clinical data informing about the skeletal phenotype and presence or absence of a neuronopathic phenotype (Table 2). Forty‐one of 51 cases had *pure MBD* presenting with skeletal features consistent with Morquio syndrome only, ten of 51 had neurologic/neurodevelopmental deficits in addition to typical skeletal features.

**Table 2:** Clinical and biochemical features of 51 cases with *GLB1*‐related Morquio‐B disease with informative clinical data, including 13 cases with undetermined (ND) genotype (first row) and 38 cases with known genotype (subsequent rows)

Allele	References	Genotype	N	Pure MBD	MBD Plus	Corneal Clouding	Cardiac finding	Organo‐ megaly	U‐Keratane sulfate	U‐Oligo/ GAG	β‐Gal activity*
ND	[18,20,22,23,24,26,29,31,66]	‐	13	12	1	7	0	0	8	11	0‐77 (3.0)
Homozygous Pure MD & MBD Plus	[13] Case#1‐12	W273L	12	12	0	3	0	1	2	2	1.3‐10 (3.2)
	[14] Case#MB2 CV6	R201H	1	1	0	‐	‐	1	‐	‐	2.4
	[17] Case#2,3 (CV1) [21] Case#3	G438E	3	0	3	1	2	0	1	1	2.7‐8.7 (5.7)
	[15,16] Case#1,2 CV2	Y333C	2	0	2	1	1	‐	2	2	3.1‐3.4
Compound Heterozygous Pure MBD	[13] Case#13,14 11 Case#24	W273L Spl?#	3	3	0	‐	0	0	‐	‐	1.3‐1.5 (1.3)
	[11] Case#22	W273L P397A	1	1	0	‐	0	0	‐	‐	2.6
	[11] Case#23	W273L D198Y	1	1	0	‐	0	0	‐	‐	3.7
	P1	W273L N484K	1	1	0	1	1	0	1	1**	5.7
	[25] Case#1	W273R H281Y	1	1	0	1	0	0	0	1	4.7
	[21] Case#1,2 (family1)	T500A N484K	2	2	0	0	0	0	2	0	1.9‐2.1
	[13] Case#15 [11] Case#25	T500A Q408P	2	2	0	‐	‐	1	‐	‐	1.3‐1.3
	[27] Case#1	R482C Y43H	1	1	0	0	0	0	1	1	9.6
	[Paschke Unpublished]	T500A R148C	1	1	0	‐	‐	‐	‐	‐	‐
	[30] Case#1	G123R L5HfsX29	1	1	0	0	1	0	1	1	1.5
Compound Heterozygous MBD Plus	[19] Case#1,2,3 CV4a,b,c	T500A G526GfsX5	3	2	1	‐	‐	‐	‐	‐	2.4‐4.0 (4.0)
	[13] Case#16 CV3	T82M Y270D	1	0	1	‐	‐	‐	‐	‐	3.4
	[13] Case#17	R201H H281Y	1	0	1	‐	‐	‐	‐	‐	5.4
	[11] Case#21 CV5	R201H S149F	1	0	1	‐	0	0	0	‐	3.6

*Notes*: MBD Plus includes developmental delay/intellectual disability and/or neurologic findings such as epilepsy, spasticity, dystonia.

*Abbreviations*: Case# = the number of the case described in the respective literature reference; CV = case vignette; Spl? = base change unknown; U‐Keratansulfate = abnormal (increased) urinary keratansulfate excretion; U‐Oligo/GAG = abnormal urinary excretion of oligosaccharides & / or glycosaminoglycans; (0) = confirmed absence of symptom; (‐) = not reported; ND = not determined.

* = % of residual β‐galactosidase activity calculated from mean normal range in white blood cells or fibroblasts. Activities were measured against 4‐MU‐β‐galactosidase as synthetic substrate.

** Keratane sulfate containing oligosaccharides were additionally demonstrated upon UPLC/MS/MS‐based determination^36^

Most frequently reported skeletal features included short stature, kyphoscoliosis/platyspondyly, coxa valga, and genu valgum, odontoid hypoplasia and joint laxity/hyperextensible joints. In some cases, the skeletal features were described as progressive sponydloepiphyseal dysplasia.^18^, ^32^ Ulnar deviation of the wrist was reported in a 7‐year‐old girl^31^ and a 7‐year‐old boy.^29^ P1 had pronounced ligament instability in the ankles and wrists the latter resulting in a weak grip. The property of tooth enamel was reported in six cases: Beck et al^22^ (n = 3), Groebe et al,^24^ and van Gemund et al^32^ and was normal in all cases but one (Guvec et al 25).

Spinal canal narrowing without myelocompression was reported in a 7‐year‐old male and a 10‐year‐old female,^18^, ^31^ and in a 40‐year‐old woman with myelocompression.^23^ In a 15‐year‐old male with *pure MBD* 22 spinal malalignment had led to spastic paraplegia.

Growth parameters were reported in 21 of 51 patients (Table 3 **)**. Short stature was a constant feature in adolescents and adults, whereas most of the younger patients had body heights within 1 SD of mean. The progressive nature of growth impairment is demonstrated by longitudinal growth data available from single patients.

**Table 3:** Growth parameters in 21 patients with MBD (n = 16 *pure MBD*; n = 5 *MBD plus*)

(Case number) Reference (Ethnicity] [GLB1 variant]	Age at growth assessment	Body height (percentile %)	Body weight (percentile %)	Calculated BMI (kg/m^2^ 37) (percentile %)
***Pure MBD***				
Male				
(1) Sheth et al 2002^29^ [East Indian] [ND]	3 y	78.8 cm (0.007%, −3.8SD)	10 kg (>3%)	16.1 (75%)
(2) van Gemund et al 1983^32^ [Caucasian/Dutch] [ND]	3 y 8 mo 5 y 5 mo 8 y 3 mo 11 y 3 mo 15 y 4 mo	100.2 cm (60%, +0.2SD) 109.6 cm (28%, −0.6SD) 121.6 cm (12%, −1.2SD) 132 cm (0.06%, −3.2SD) 149.7 cm (0.5%, −3.6SD)	15.6 kg (25%‐50%)	15.5 (50%)
(3) Sohn et al 2012^30^ [Korean][L5HfsX/G123R]	6 y	112.4 cm (22%, −0.8SD)	19.9 kg (50%)	15.8 (50%)
(4) Groebe et al 1980^24^ [Caucasian/Greek] [ND]	6 y 3 mo	107 cm (1.7%, −2.1SD)	Not available	—
(5) Trojak et al 1980^31^ [Caucasian] [ND]	7 y	119 cm (29%, −0.6)	26.6 kg (80%)	18.8 (94%)
(6) Sheth et al 2002^29^ [East Indian] [ND]	7 y	94 cm (0%, −5.2SD)	14 kg (>3%)	15.8 (50%)
(7) Ishii et al 1995^27^ [Japanese] [Y83H/R482C]	11 y 7 mo	135.2 cm (5%, −1.6SD)	37.8 kg (50%)	20.7 (85%)
(8) Groebe et al 1980^24^ [Caucasian/Austrian] [W273L/W273L^a^]	25 y 5 mo	137 cm (0%, −5.4SD)	41 kg (<<3%)	21.8 (50%)
(9) (CV 5c) Paschke et al 2014^19^ [South American] [T500A/Gly526GlyfsX5]	39 y	162 cm (2.4%, −2SD)	Not available	—
(10) P1 [Caucasian/Canadian] [W273L/N484K]	5 y 8 mo 10 y 9 mo 14 y 8 mo	112 cm (34%, −0.4SD) 123 cm (0.4%, −2.7SD) 134 cm (0.001%, −4.4SD)	23 kg (75%) 27 kg (10%) 41 kg (3%)	18.3 (97%) 17.8 (75%) 22.8 (85%)
**Female**				
(11) van Gemund et al 1983^32^ [Caucasian/Dutch] [ND]	3 y 4 y 6 mo 7 y 6 mo 11 y 9 mo	95.6 cm (69%, +0.5SD) 104.8 cm (50%, ±0SD) 118.5 cm (18%, −0.9.1SD) 135.5 cm (2.6%, −1.9SD)	13.5 kg (15%) Not available Not available Not available	14.8 (≥50%) — — —
(12) van Gemund et al 1983^32^ [Caucasian/Dutch] [ND]	5 y 8 mo 7 y 5 mo 10 y 6 mo 14 y 3 mo 17 y 4 mo	112 cm (46%, −0.1SD) 118.8 cm (23%, −0.8SD) 127.5 cm (2%, −2.1SD) 134 cm (<0.004%, −3.9SD) 138.5 cm (<0.005%, −3.9SD)	22.5 kg (50%) Not available Not available Not available Not available	17.9 (90%) — — — —
(13) O'Brien et al 1976^18^ [Caucasian/Italian] [ND]	12 y	143.5 cm (15%, −1SD)	41 kg (50%)	19.9 (50%)
(14) Arbisser et al 1977^20^ [Caucasian?] [ND]	14 y	138.5 cm (0.09%, −3.1SD)	Not available	
(15) Gucev et al 2008^25^ [Caucasian/Macedonian] [W273R/H281Y]	24 y	138 cm (0.007%, −3.8SD)	Not available	—
(16) Di Cesare et al 2012^23^ [Caucasian/Italian] [ND]	43 y	150 cm (3.9%, −1.8SD)	Not available	—
***MBD plus***				
**Male**				
(17) Giugliani et al 1987^15^; Mayer et al 2009^16^ [Arabic] [Y333C/Y333C]	11 y	114 cm (0.002%, −4.1SD)	Not available	—
(18) (CV 5b) Paschke et al 2014^19^ [South American] [T500A/Gly526GlyfsX5]	30 y	155 cm (12%, −1.2SD)	Not available	—
**Female**				
(19) Giugliani et al 1987^15^; Mayer et al 2009^16^ [Arabic] [Y333C/Y333C]	8 y	118 cm (8%, −1.4SD)	Not available	—
(20) Bagshaw et al 2002^21^ [ND] [N484K/T500A]	18 y	147 cm (0.8%, −2.4SD)	Not available	—
(21) Roze et al 2005^17^ [Romanian] [G438E/G438E]	19 y	140 cm (0.03%, −3.4SD)	Not available	—

*Note*: Percentile value obtained from https://tall.life/height-percentile-calculator-age-country/ and CDC Weight for Age Percentiles.

Abbreviation: ND, not determined.

a GLB1 mutation published in Paschke et al.^13^

Corneal clouding and cardiac valve pathology were reported in 20 of 51 cases (Table 2). Hepato‐splenomegaly, was reported in only two of 51 MBD cases (n = 1 homozygous W273L [case 2: Groebe et al^24^ and Paschke et al^13^]; n = 1 homozygous R201H [MB2 Santamaria et al^14^ *= CV 6]*).

Abnormal urinary excretion of glycosaminoglycans and of oligosaccharide containing glycoproteins was inconsistently reported. In P1 urinary oligosaccharides showed an abnormal band on thin layer chromatography and glycosaminoglycan excretion was mildly elevated (18 mg/mmol creatinine; reference range < 15) (Cetylpyridinium chloride test). Keratan sulfate was undetectable on urinary glycosaminoglycan electrophoresis, but keratan sulfate disaccharides were clearly elevated (28.5 μg/mg creatinine; reference range 0.24‐2.71) upon UPLC/MS/MS‐based determination.^36^

Ultrastructural examination of a skin biopsy was reported in one single case^18^ showing interstitial and cytoplasmatic U‐shaped lamellar inclusions but absence of lysosomal inclusions found in GM1 gangliosidosis.^38^ In Holzgreve et al^39^ description of patients with Morquio syndrome, Adler‐Reilly granular abnormalities were found in blood smears of patients with Morquio‐A disease but not in the those with MBD.

Quantitative values of β‐galactosidase activities were available in 49 of 51 cases with informative clinical data (β‐galactosidase was measured but activity was not reported in the case published by Di Cesare et al^23^; β‐galactosidase was not measured in one of three siblings with MBD published by van Gemund et al^32^). β‐galactosidase activities were given in nmol/mg/min or in nmol/mg/min and/or as percent of normal. For reasons of comparability we calculated the percentage of the residual activities based on the respective mean of the normal range for all cases. Activities were measured either in white blood cells or in fibroblasts. Overall, residual β‐galactosidase activities ranged between 0% and 17% in *pure MBD* cases and between 2% and 8.7% in *MBD plus* cases (Table 2).

In 47 of 63 cases genotypic information was available, harboring 28 different *GLB1* variants. The most frequent alleles were W273L (34/94 alleles) and T500A (11/94 alleles). The characteristics of the 28 variants are shown in Table 4. Twenty‐five of 28 variants were missense, two were frameshift, and one was splice site. Seven variants found in the reviewed cases had previously been tested for chaperone sensitivity with three of them being reported chaperone sensitive (T82M, R201H, H281Y).

**Table 4:** Characteristics and chaperone sensitivity (as reported in the literature) of 28 *GLB1* mutant alleles identified in 47 cases with Morquio‐B phenotype and reported genotype

Mutant allele	Number of alleles		Clinical phenotype		Exon AA residue location	Base change	Impact on translated GLB1 allele *(missense*, *nonsense*, *frame shift)*	Mechanism of GLB1 deficiency (*complete absence*, *premature degradation*, *catalytic*, *unknown)*	Amenability to chaperon therapy and degree of β‐galactosidase rescue activity (r*eference*)
	HMZ	HTZ	MBD *pure* (n = number of cases)	MBD *plus* (n = number of cases)					
L5HfsX29	0	1	1	0	1 Signal peptide	c.13_14insA	Frameshift	Truncated protein that lacks most domains	Unlikely
T82M	0	1	0	1	2 TIMBD	c.245C>T	Missense	Premature degradation	Sensitive^69^
spl?	0	3	3	0	3 —	c.246G>T	Splice site defect	Inactive two major products lacking exon 2 and exons 2‐5	Unlikely
Y83C/D441	0	1	ui	ui	3 TIMBD	c.248A>G	Missense	Affects ligand recognition	Unlikely
Y83H	0	1	1	0	3 TIMBD	c.247T>C	Missense	Affects ligand recognition	Unlikely
G123R	0	1	1	0	3 TIMBD	c.367G>A	Missense	Complete absence	Unlikely
R148C	0	1	1	0	4 TIMBD	c.442C>T	Missense	Complete absence	Unlikely
S149F	0	1	0	1	4 TIMBD	c.446C>T	Missense	Unknown	Insensitive *(Ph[TFM]2OHex‐DGJ) 7.2‐fold; 21.0%^40^ *[TFM]3OHex‐DGJ 7.1‐fold; 20.7% 40
L173P/T500A	0	2	ui	ui	5 TIMBD	c.518 T>C	Missense	Complete absence	Unlikely
D198Y	0	1	1	0	6 TIMBD	c.592G>T	Missense	Located on protein surface and leads to reduced activity	Unknown
R201H	2	2	1	2	6 TIMBD	c.602G>A	Missense	Premature degradation	Sensitive *(DLHex‐DGJ), 11.1‐12.5‐fold; 27.3%‐33.9% 41 *(Ph[TFM]2OHex‐DGJ) 7.2‐9.1‐fold; 16.7%‐21.0% 40 *[TFM]3OHex‐DGJ 7.1‐10.0‐fold; 18.3%‐20.7% 40
H281Y	0	2	1	1	8 TIMBD (catres)	c.841C>T	Missense	Catalytic	Sensitive *(DLHex‐DGJ), 12.5‐fold; 30.4%^41^
W273L	24	10	18 ui (n = 16)	0	8 TIMBD (catres)	c.817TG>CT	Missense	Catalytic	Insensitive *(DLHex‐DGJ), 1.3‐fold; 5.1%^41^ *(Ph(TFM)2OHex‐DGJ) 1.2‐fold; 2.1% 40 *(TFM)3OHex‐DGJ 1.4‐fold; 2.5%^40^
W273R	0	1	1	0	8 TIMBD (catres)	817T>C	Missense	Catalytic	Unlikely
Y270D	0	1	0	1	8 TIMBD (catres)	c.808T>G	Missense	Catalytic	Insensitive *(DLHex‐DGJ), 1.7‐fold; 0.4%^41^
Y333C	4	0	0	2	10 TIMBD (catres)	c.998 A>G	Missense	Catalytic	Unlikely
P397A	0	1	1	0	12 End of TIMbeta1 loop	c.1189C>G	Missense	Premature degradation	Unknown
Q408P	0	2	2	0	12 Beta domain 1	c.1223A>C	Missense	unknown	Unknown
D441N	0	1	ui	ui	13 Beta domain 1	c.1321G>A	Missense	unknown	Unknown
G438E	6	0	3	0	13 Beta domain 1	c.1313G>A	Missense	Reduced activity	Insensitive *(DLHex‐DGJ), 2.3‐fold; 16.4%^41^ *(Ph(TFM)2OHex‐DGJ) 1.3‐fold; 7.3% 40 *(TFM)3OHex‐DGJ 1.3‐fold; 7.1% 40
Y444C	0	1	ui	ui	13 Beta domain 1	c.1331A>G	Missense	Reduced activity	Unknown
N484K	0	4	4	0	14 Beta domain 1	c.1452C>A	Missense	unknown	Unknown
R482C	0	1	1	0	14 Beta domain 1	c.1444C>T	Missense	Complete absence	Unknown
R482H	0	3	ui	ui	14 Beta domain 1	c.1445G>A	Missense	Complete absence	Unknown
G494S	0	1	ui	ui	15 Beta domain 1	c.1480G>A	Missense	Complete absence	Unknown
T500A N = 4	0	11	5 ui (n = 4)	2	15 Beta domain 1	c.1498A>G	Missense	Possible catalytic	Unknown
G526GfsX5	0	3	1	2	15 Beta domain 1	c.1577dupG	Frame shift	Truncated and inactive gene product lacking a functionally essential domain in exon 16	Unknown
W509C	0	1	ui	ui	15 Beta domain 1	c.1527G>T	Missense	Unknown	Unknown
**Total # alleles**	36	58							
**Total # cases**	18	29							

Abbreviations: *, name of chaperone; %, percent of normal activity; catres, adjacent to catalytic residue; fold, −fold increase of baseline activity; HMZ, homozygous; HTZ, heterozygous; TIMBD, TIM barrel domain; ui, uninformative clinical data.

Information about the clinical (skeletal and neuronopathic) phenotype in conjunction with the underlying *GLB1* mutations was available in 38 cases (Table 2). Twenty‐nine of 38 had *pure MBD* (dysostosis multiplex type Morquio syndrome without evidence of neuropathic involvement). Additional neuronopathic manifestations (*MBD plus*) were reported 10 of 51 cases (nine of 38 with known genotype and one of 13 with unknown genotype) (Table 2). Neuronopathic manifestations included: developmental delay/intellectual disability, loss of motor and cognitive skills with onset in late infancy, delayed/impaired speech, ataxia, spasticity, dystonia, myoclonia, choreoatetosis. Brain MRI was reported in only one of the *MBD plus* cases (Roze et al,^17^ case 2) and was normal. These 38 cases harbored 21 of the 28 *GLB1* variants identified in this review.

Four of 21 variants were present in homozygosity: W273L (12 cases/five families); R201H (one case/one family); G438E (three cases/two families); Y333C (two cases/one family). Homozygous W273L was invariably associated with *pure MBD*. *Pure MBD* was also reported in a unique case homozygous for the R201H allele, in those seven individual compound heterozygous for W273L and W273R, respectively, and in six of the eight cases reported as compound heterozygous for T500A. Patients homozygous for G438E and for Y333C showed neuronopathic features including cerebral neurologic and developmental involvement.

To demonstrate the clinical heterogeneity of *MBD plus* disease we extracted the following case vignettes from the literature reviewed:

CV 1 (G438E/G438E) (Roze et al,^17^ case 2) features an MBD plus phenotype, characterized by early onset developmental delay, intellectual disability, and subsequent progressive dystonia. A 19‐year‐old woman presented with a history of normal walking at age of 2.5 year and gradual loss of motor performance at age of 7 to ‐8 years and severe gait disturbances at age 17 years with an ability to walk unsupported for 10 to 20 m only. Neurologically she had generalized dystonia with facial grimacing, dysarthria, swallowing difficulties, drooling, and choreoathetoid movements and myoclonic jerks. She had moderate intellectual impairment.

CV 2 and CV 3 (Y333C/Y333C (case 1, Giugliani et al^15^ and Mayer et al^16^) and (T82M/Y270D) (case 16 in Paschke et al^13^) feature an MBD plus phenotype, characterized by unremarkable early development and progressive spasticity and speech impairment later on. CV 2 was able to walk and had normal speech development at age 18 months. At age 11, he presented with speech ability limited to a few words and inability to walk. He had an increased tone of the limbs and brisk tendon reflexes. CV 3 (male) was diagnosed with MBD based on characteristic skeletal abnormalities at the age of 4. While at the age of diagnosis his motor and cognitive development was normal, he gradually lost his ability to speak and he became tetraspastic after the age of 10 years.

CV 4a, 4b, 4c (T500A/c.1577dupG = p.G526GfsX5)^19^ demonstrate the phenotypic variability of the same genotype. Among three male unrelated patients with the same genotype, two (ages 7 and 39) had skeletal features consistent with MBD without neuronopathic involvement, while one (age 31) had psychomotor delay and speech difficulties at age 3 and thereafter developed borderline intelligence and neurological regression. ß‐galactosidase activities were not discriminative (4% in both cases with *pure MBD* and 2% in the *MBD plus* case).

CV 5 (R201H/S149F) (case 21 in Hofer et al^11^) features an unspecific (non‐Morquio) type of dystostosis multiplex, associated with mild intellectual disability. This 14‐year‐old male was described as “atypical” MBD. He presented with mild intellectual disability and dorsolumbar kyphoscoliosis, but characteristic Morquio features (short, disproportionate stature, dysplasia of the odontoid, hip dysplasia, genua valga) were not present when assessed at 19 years. Corneal clouding, cherry red spots, cardiac involvement, and organomegaly were absent.

CV 6 (R201H/R201H) (MBD2 Santamaria et al^14^) features the sole described patient with pure MBD who is homozygous for a variant that previously has been described in association with neuronopathic (types 2 and 3) GM1 gangliosidosis. At the age of 16, this male patient exhibited skeletal features characteristic of MBD. He had a history of a normal development, his cognition was within normal range and neurologic signs and symptoms suggestive of a primary neuronopathic course were absent.

## 3 DISCUSSION

### 3.1 MBD phenotypes

The results of this study have reinforced the general understanding that MBD is a distinct variant of *GLB1*‐related disease with an axial and appendicular dysostosis multiplex as initially described in *GALNS*‐related Morquio‐A disease. However, while Morquio‐A disease is invariably associated with normal intellectual development and lifelong absence of primary neuropathic manifestations, only just below 80% of the *GLB1*‐related MBD cases presented with a pure skeletal phenotype. The remaining 10 cases showed additional primary neuronopathic manifestations. These findings indicate that *GLB1*‐related MBD occurs in two forms: *pure MBD* and *MBD plus* neuronopathic manifestations.

Results also have reinforced the general understanding that the skeletal manifestations are mild in MBD when compared to typical Morquio‐A disease and clinically indistinguishable from mild Morquio‐A variants.^39^, ^42^, ^43^ Along with this notion, we previously could show that the height of adult MBD patients is significantly less compromised than of those with typical Morquio‐A disease.^44^ Notably, three of the five adults (24‐39 years old) depicted in Table 3 had only a mildly impaired body height (minus 1.2 SD‐minus 2 SD). Conversely, among the six individuals younger than 6 years, only the patient reported by Sheth et al^29^ had early onset dwarfism with a body height at minus 3.8 SD at the age of 3 years.

In cases with mild skeletal involvement, such as CV 5 who was described as “atypical Morquio‐B” a distinction between MBD and GM1‐gangliosidosis with unspecific dysostosis may be challenging. Therefore, for the sake of a precise classification, we recommend that the diagnosis of MBD should be assigned only if there are ≥3 radiological findings characteristic of Morquio syndrome, such as platyspondyly and vertebral beaking involving all segments of the spine, odontoid hypoplasia, epi‐and metaphyseal dysplasia of long bones, and hip dysplasia.

Other manifestations, which are typically observed in *GALNS*‐related Morquio‐A disease^45^, ^46^, ^47^ were not reported at all (such as atlantoaxial subluxation, hearing impairment, tracheal stenosis) or were reported only in a minority of cases (such as spinal cord narrowing, myelocompression, hepatosplenomegaly, cardiac valve pathology, tooth enamel abnormalities). Cardiomyopathy and retinal cherry red spot, which typically occur in GM1‐gangliosidosis,^1^ were not reported in any of the MBD cases. Corneal clouding was observed mainly in cases who had mutations in the catalytic domain essential for keratan sulfate substrate processing such as W273L and Y333C.^4^, ^10^, ^33^, ^48^

Impaired elastogenesis has been shown in GM1 gangliosidosis and MBD.^33^, ^49^, ^50^ Apart from a single study in a skin biopsy of a patient with MBD,^18^ biochemical and morphologic studies on extracellular matrix or bone pathology have not been performed for MBD. Future studies similar to those performed for numerous other LSDs^51^ and for Morquio‐A disease^52^, ^53^ may serve for a better understanding of the clinical differences between Morquio‐A disease and MBD (eg, why surgical operations in MBD patients differ significantly from those with Morquio‐A disease both in regards to the types of intervention, and in regards to the age at which these surgeries become necessary.^44^

Neuronopathic manifestations in MBD span from an early onset global developmental delay with delayed achievement of motor milestones, speech delay to intellectual disability, progressive spasticity, and dystonia (CV 1). Onset of neurocognitive deficiencies can be later in life (CV 2), with skeletal findings being the first red flag (CV 3). Clinicians should bear in mind that patients diagnosed with *pure MBD* in early childhood might develop neuronopathic problems later on.

### 3.2 MBD genotypes

W273 L was invariably associated with *pure MBD*. The amino acid residue Trp‐273 resides at the entrance of the ligand‐binding pocket of β‐galactosidase, which acts as a holder of substrates for catalytic reaction. W273L affects the degradation of keratan sulfate more severely than the turnover of GM1‐ganglioside, explaining the predominance of skeletal manifestations.^7^, ^10^, ^11^, ^12^, ^34^, ^54^, ^55^

*Pure MBD* also was reported in single a case homozygous for R201H (CV 6). Arg‐201 is located on the lateral face of the TIM barrel domain, which is far from the ligand‐binding pocket^7^, ^56^ thus not specifically affecting the catalytic activity towards keratan sulfate. It has rather been suggested that the R201H mutation results in a mislocalized, unstable precursor protein.^11^, ^41^ Several cases were found where the R201H allele was associated with type 2 GM1‐gangliosidosis,^1^ while its association with *pure MBD* 12 remains to be confirmed in more cases.

The other *GLB1* variants found in homozygosity (G438E and Y333C), were associated with *MBD plus* (Table 2). G438E causes an abnormal complex formation alone or coupled with keratan sulfate binding^21^ with a relatively high (6.1%) residual activity.^11^ Results of enzyme activity assays using different substrates suggest that Y333, similar to W273L, affects the active site of β‐galactosidase rather than affecting the enzyme stability^16^ comparable to D332, the adjacent amino acid residue, which is directly involved in the catalytic reaction.^11^ Y333H is not invariably associated with MBD, as homozygous cases have been described with Type 2 GM1‐gangliosidosis lacking the specific features of Morquio syndrome.^11^

After W273L, T500A was the second frequent allele occurring in heterozygosity in 11/58 alleles. In the 38 cases with clinical and genetic information, six of eight compound heterozygous cases presented with *pure MBD*.

### 3.3 β‐galactosidase activity and biomarkers

We were not able to establish a correlation between residual β‐galactosidase activities, genotypes and phenotypes. The main reason for the inability to discriminate molecular characteristics of the various *GLB1* mutations is the use of synthetic substrates (eg, 4‐MU‐β‐galactoside) for the determination of β‐galactosidase activity, which only allows a rough discrimination between zero residual activities (eg, in infantile GM1‐gangliosidosis), and activities up to 2%‐10% (eg, in late onset GM1‐gangliosidosis and MBD).^11^ To precisely determine the biochemical characteristics of β‐galactosidase mutants, measurements using natural substrates are needed. However, such measurements are laborious and have rarely been performed.^54^, ^55^ Technical variations in the enzyme assays across the various labs and the type (white blood cells, fibroblasts) and quality of samples used also contribute to the poor correlation of β‐galactosidase activity with the genotype.

Likewise, limited information is has been found regarding a correlation between chemical biomarkers and the genotype. Keratan sulfate is the main storage product in MBD, however analytical challenges imposed by the use of traditional methods may explain why in the cases reviewed here keratan sulfate was either not determined or information was mostly restricted to its presence or absence. Quantitative measurements of keratan sulfate using LC‐MS/MS‐based technologies have only recently become available. As shown in P1, urinary keratan sulfate accumulation could only be shown upon LC‐MS/MS‐based analysis but not upon traditional glycosaminoglycan electrophoresis. Other studies employing LC‐MS/MS‐based technology have shown an accumulation of mono‐ and disulfated keratan sulfate species in blood and urine of single MBD patients^57^ and a correlation with clinical severity has been shown in Morquio‐A patients.^45^ Further studies are needed to determine age and phenotype related biomarker profiles in MBD patients.

### 3.4 Chaperone sensitivity

Several pharmacological chaperones acting on β‐galactosidase including galactose, N‐octyl‐4‐epi‐beta‐valienamine (NOEV), alkylated or fluorinated derivates of desoxynojirimycine (DGJ), and (5aR)‐5a‐C‐Pentyl‐4‐epiisofagomine have been tested against numerous *GLB1* mutant enzymes.^58^, ^59^, ^60^, ^61^, ^62^

As a general rule, chaperone responsive mutant proteins harbor intact catalytic sites but fail in achieving full maturation or appropriate localization in the lysosomes due to protein misfolding or lack of protection by protective protein/cathepsin A.^37^, ^40^, ^41^, ^63^ Three of the *GLB1* alleles identified in this review (T82M, R201H, H281Y) have been shown in the literature to be chaperone responsive. The most pronounced response was observed in the R201H allele using DGJ derivatives as chemical chaperones. Human fibroblasts carrying this variant in homo‐ or heterozygosity showed an up to 12.5‐fold increase of basal β‐galactosidase activity resulting in 30% of normal control activity.^40^, ^41^ According to theoretical considerations^64^ and evidence shown in cell cultures,^63^, ^65^ residual enzyme activities beyond 10% to 15% may be sufficient to avoid substrate accumulation. A comparable magnitude of β‐galactosidase enhancement has been reported by^59^ in 10 out of 15 *GLB1*‐deficient fibroblast lines tested against a 4‐*epi*‐isofagomine derivative. Interestingly, six fibroblast lines carried at least one mutation at the amino acid residue Arg‐201. Despite significant achievements in preclinical research, with the exception of the aminosugar Miglustat,^66^ chaperone therapy yet has not been established for patients with *GLB1*‐related conditions.

W273L, the most frequent MBD allele, is not sensitive to chaperon rescue as it encodes for a catalytic mutant within an otherwise stable, normally trafficked and localized protein^12^, ^40^, ^67^ *(Paschke unpublished)*. Therefore only compound heterozygous individuals harboring a second chaperone‐sensitive allele will benefit from this form of therapy. Substantial progress in the development of gene therapies for *GLB1*‐related conditions^68^ will benefit patients with variants not amenable to chaperone therapies.

## 4 CONCLUSION AND OUTLOOK

Overall, this review of published cases with MBD has shown that MBD occurs as a spectrum of distinct skeletal and non‐skeletal (neuronopathic) manifestations.

While there is a clear association between *pure MBD* and the W273L allele, further studies are needed to better determine genotype‐phenotype correlations of *MBD plus* alleles as well as their role in elastogenesis and bone pathology.

Careful clinical phenotyping of this ultra‐rare condition is important for elucidation of the natural history of MBD informing the choice of outcomes in future clinical trials. Clinical assessments should include a full skeletal survey with additional attention to the cranio‐cervical junction, as well as a full clinical, neurologic, and neurocognitive exams, including a brain MRI. Biochemical phenotyping should include the determination of β‐galactosidase activity in white blood cells or fibroblasts, as well as quantitative (LC‐MS/MS based) determination of urinary glycosaminoglycans and keratan sulfate‐derived oligosaccharides. We have started collecting data via an international patient registry^8^ and are currently initiating repositories for longitudinal data and biological sample collection.

## CONFLICT OF INTEREST

S.S.‐I. holds The Priest Family Fund for Morquio‐B Research, a UBC‐based stewardship grant. She has received educational grants from Biomarin, Shire, Recordati and she serves/served as PI in clinical trials and postmarketing registries sponsored by Actelion, Biomarin, Shire, Ultragenyx. I.A., N.Y., and E.P. have no conflicts to declare.

## AUTHOR CONTRIBUTIONS

I.SA. performed the literature review, extracted and analyzed data, and wrote the manuscript. N.Y. coordinated Morquio‐B related research, participated in manuscript writing and editing. E.P. contributed and critically reviewed data and biochemical/genetic data. S.S.‐I. initiated this research project, and supervised progress of work and data analysis, analyzed data, contributed to manuscript writing, and edited the final version of the manuscript.

## ETHICS APPROVAL

Not applicable.

## PATIENT CONSENT

Obtained from patient 1 (P1).

## References

[1] Regier DS , Tifft CJ . GLB1‐related disorders In: Adam MP, Ardinger HH, Pagon RA, et al., eds. GeneReviews® [Internet]. Seattle, WA: University of Washington, Seattle; 2013:1993‐2018. 24156116

[2] Bonafe L , Cormier‐Daire V , Hall C , et al. Nosology and classification of genetic skeletal disorders: 2015 revision. Am J Med Genet A. 2015;167A(12):2869‐2892. 26394607 10.1002/ajmg.a.37365

[3] Spranger JW , Brill PM , Nishimura G , Superti‐Furga A , Unger S . Bone Dysplasias. An Atlas of Genetic Disorders of Skeletal Development. 3rd ed New York: Oxford University Press; 2012:565‐594.

[4] Suzuki Y , Nanba E , Matsuda J , Higaki K , Oshima A . β‐Galactosidase deficiency (β‐galactosidosis): GM1 gangliosidosis and Morquio‐B disease In: Beaudet AL, Vogelstein B, Kinzler KW, et al., eds. The Online Metabolic and Molecular Bases of Inherited Disease [Internet]. New York, NY: The McGraw‐Hill Companies, Inc.; 2014.

[5] Morquio L . Sur une forme de dystrophie osseuse familiale. Bull Soc Pediatr. 1929;27(2):145‐152.

[6] Brailsford JF . The classics: chondro‐osteo‐dystrophy. Roentgenographic and clinical features of a child with dislocation of vertebrae. Am J Surg. 1929;7:404. 770041

[7] Ohto U , Usui K , Ochi T , Yuki K , Satow Y , Shimizu T . Crystal structure of human beta‐galactosidase: structural basis of G M1 gangliosidosis and morquio‐B diseases. J Biol Chem. 2012;287(3):1801‐1812. 22128166 10.1074/jbc.M111.293795 PMC3265862

[8] Morreau H , Galjart NJ , Gillemans N , Willemsen R , van der Horst GT , d'Azzo A . Alternative splicing of beta‐galactosidase mRNA generates the classic lysosomal enzyme and a beta‐galactosidase‐related protein. J Biol Chem. 1989;264(34):20655‐20663. 2511208

[9] Privitera S , Prody CA , Callahan JW , Hinek A . The 67‐kDa enzymatically inactive alternatively spliced variant of β‐ galactosidase is identical to the elastin/laminin‐binding protein. J Biol Chem. 1998;273(11):6319‐6326. 9497360 10.1074/jbc.273.11.6319

[10] Callahan JW . Molecular basis of GM1 gangliosidosis and Morquio disease, type B. structure‐function studies of lysosomal beta‐galactosidase and the non‐lysosomal beta‐galactosidase‐like protein. Biochim Biophys Acta. 1999;1455(2–3):85‐103. 10571006 10.1016/s0925-4439(99)00075-7

[11] Hofer D , Paul K , Fantur K , et al. GM1 gangliosidosis and Morquio‐B disease: expression analysis of missense mutations affecting the catalytic site of acid beta‐galactosidase. Hum Mutat. 2009;30(8):1214‐1221. 19472408 10.1002/humu.21031

[12] Hofer D , Paul K , Fantur K , et al. Phenotype determining alleles in GM1 gangliosidosis patients bearing novel GLB1 mutations. Clin Genet. 2010;78(3):236‐246. 20175788 10.1111/j.1399-0004.2010.01379.x

[13] Paschke E , Milos I , Kreimer‐Erlacher H , et al. Mutation analyses in 17 patients with deficiency in acid ‐galactosidase : three novel point mutations and high correlation of mutation W273L with Morquio disease type B. Hum Genet. 2001 Aug;109(2):159‐166. 11511921 10.1007/s004390100570

[14] Santamaria R , Chabás A , Coll MJ , Miranda CS , Vilageliu L , Grinberg D . Twenty‐one novel mutations in the GLB1 gene identified in a large group of GM1‐gangliosidosis and Morquio‐B patients: possible common origin for the prevalent p.R59H mutation among gypsies. Hum Mutat. 2006;27(10):1060. 10.1002/humu.9451 16941474

[15] Giugliani R , Jackson M , Skinner SJ , et al. Progressive mental regression in siblings with Morquio disease type B (mucopolysaccharidosis IV B). Clin Genet. 1987;32(5):313‐325. 3121219 10.1111/j.1399-0004.1987.tb03296.x

[16] Mayer FQ , Pereira FDS , Fensom AH , Slade C , Matte U , Giugliani R . New GLB1 mutation in siblings with Morquio type B disease presenting with mental regression. Mol Genet Metab. 2009;96:148. 19091613 10.1016/j.ymgme.2008.11.159

[17] Roze E , Paschke E , Lopez N , et al. Dystonia and parkinsonism in GM1 type 3 gangliosidosis. Mov Disord. 2005;20(10):1366‐1369. 15986423 10.1002/mds.20593

[18] O'Brien JS , Gugler E , Giedion SA , et al. SpondyloepiphyseaI dysplasia, corneal clouding, normal intelligence and acid beta‐galactosidase deficiency. Clin Genet. 1976 May;9(5):495‐504. 817853 10.1111/j.1399-0004.1976.tb01603.x

[19] Paschke E , Giugliani R , Marques Lourenco C , Windischhofer W . Mutation p.500T>a in exon 10 of the GLB1 gene is not prognostic for Morquio‐B disease. J Inborn Errors Metabo Screen. 2014;2.

[20] Arbisser AI , Donnelly KA , Scott CIJ , et al. Morquio‐like syndrome with beta galactosidase deficiency and normal hexosamine sulfatase activity: mucopolysacchariodosis IVB. Am J Med Genet. 1977;1(2):195‐205. 416714 10.1002/ajmg.1320010205

[21] Bagshaw RD , Zhang S , Hinek A , et al. Novel mutations (Asn 484 Lys, Thr 500 Ala, Gly 438 Glu) in Morquio‐B disease. Biochim Biophys Acta. 2002;1588(3):247‐253. 12393180 10.1016/s0925-4439(02)00172-2

[22] Beck M , Petersen EM , Spranger J , Beighton P . Morquio's disease type B (beta‐galactosidase deficiency) in three siblings. S Afr Med J. 1987;72(10):704‐707. 3120323

[23] Di Cesare A , Di Cagno A , Moffa S , Teresa P , Luca I , Giombini A . A description of skeletal manifestation in adult case of Morquio syndrome: radiographic and MRI appearance. Case Rep Med. 2012;2012:324596. 22829837 10.1155/2012/324596 PMC3398650

[24] Groebe H , Krins M , Schmidberger H , et al. Morquio syndrome (mucopolysaccharidosis IV B) associated with beta‐galactosidase deficiency. Report of two cases. Am J Hum Genet. 1980;32(2):258‐272. 6446239 PMC1686002

[25] Gucev ZS , Tasic V , Jancevska A , et al. Novel beta‐galactosidase gene mutation p.W273R in a woman with mucopolysaccharidosis type IVB (Morquio‐B) and lack of response to in vitro chaperone treatment of her skin fibroblasts. Am J Med Genet Part A. 2008;146(13):1736‐1740. 10.1002/ajmg.a.32318 18546276

[26] Holzgreve W , Grobe H , Figura K Von , Kresse H , Beck H , Mattei JF . Morquio Syndrome. Clinical findings in 11 patients with MPS IVA and 2 patients with MPS IVB. Hum Genet. 1981;57(4):360‐365. 6793501 10.1007/BF00281685

[27] Ishii N , Oohira T , Oshima A , et al. Clinical and molecular analysis of a Japanese boy with Morquio‐B disease. Clin Genet. 1995;48:103‐108. 7586649 10.1111/j.1399-0004.1995.tb04065.x

[28] Maroteaux P , Stanescu V , Stanescu R , Kresse H , Hors‐Cayla MC . Heterogeneity of formes frustes of Morquio's disease. Arch Fr Pediatr. 1982;39(Suppl 2):761‐765. 6219649

[29] Sheth JJ , Sheth FJ , Bhattacharya R . Morquio‐B syndrome (MPS‐IV B) associated with galactosidase deficiency in two siblings. Indian J Pediatr. 2002;69(79):109‐111. 11876111 10.1007/BF02723790

[30] Sohn YB , Park HD , Park SW , et al. A Korean patient with Morquio‐B disease with a novel c.13_14insA mutation in the GLB1 gene. Ann Clin Lab Sci. 2012;42(1):89‐93. 22371915

[31] Trojak JE , Ho CK , Roesel RA , et al. Morquio‐like syndrome (MPS IV B) associated with deficiency of a β‐galactosidase. Johns Hopkins Med J. 1980;146(2):75‐79. 6766519

[32] van Gemund JJ , Giesberts MAH , Eerdmans RF , Blom W , Kleijer WJ . Morquio‐B disease, spondyloepiphyseal dysplasia associated with acid galactosidase deficiency. Report of three cases in one family. Hum Genet. 1983;64(1):50‐54. 6409799 10.1007/BF00289478

[33] Hinek A , Zhang S , Smith AC , Callahan JW . Impaired elastic‐fiber assembly by fibroblasts from patients with either Morquio‐B disease or infantile GM1‐gangliosidosis is linked to deficiency in the 67‐kD spliced variant of b‐galactosidase. Am J Hum Genet. 2000;67:23‐36. 10841810 10.1086/302968 PMC1287082

[34] Oshima A , Yoshida K , Shimmoto M , Fukuhara Y , Sakuraba H , Suzuki Y . Human beta‐galactosidase gene mutations in Morquio‐B disease. Am J Hum Genet. 1991;49(5):1091‐1093. 1928092 PMC1683264

[35] Pronicka E , Tylki A , Czartoryska B , Górska D . Three cases of beta‐galactosidase deficiency. Klin Pädiatrie. 1981;193(4):343‐346. 10.1055/s-2008-1034492 6790814

[36] Auray‐Blais C , Lavoie P , Maranda B , Boutin M . Evaluation of urinary keratan sulfate disaccharides in MPS IVA patients using UPLC‐MS/MS. Bioanalysis. 2016; Feb;8(3):179‐191. 26805456 10.4155/bio.15.239

[37] Arif M , Higaki K , Shinpo M , Nanba E . Chemical chaperone treatment for galactosialidosis: effect of NOEV on b ‐galactosidase activities in fibroblasts. Jpn Soc Child Neurol. 2016;38(2):175‐180. 10.1016/j.braindev.2015.07.006 26259553

[38] O'Brien JS , Bernett J , Veath ML , Paa D . Lysosomal storage disorders. Diagnosis by ultrastructural examination of skin biopsy specimens. Arch Neurol. 1975 Sep;32(9):592‐599. 809024 10.1001/archneur.1975.00490510048002

[39] Holzgreve W , Grobe H , Von Figura K , Kresse H , Beck H , Mattei JF . Morquio syndrome clinical findings in 11 patients with MPS IVA and 2 patients with MPS IV B. Hum Genet. 1981;57(4):360‐365. 6793501 10.1007/BF00281685

[40] Fantur KM , Wrodnigg TM , Stütz AE , Pabst BM , Paschke E . Fluorous iminoalditols act as effective pharmacological chaperones against gene products from GLB1 alleles causing GM1‐gangliosidosis and Morquio‐B disease. J Inherit Metab Dis. 2012;35:495‐503. 22033734 10.1007/s10545-011-9409-2

[41] Fantur K , Hofer D , Schitter G , et al. DLHex‐DGJ, a novel derivative of 1‐deoxygalactonojirimycin with pharmacological chaperone activity in human GM1‐gangliosidosis fibroblasts. Mol Genet Metab. 2010;100(3):262‐268. 20409738 10.1016/j.ymgme.2010.03.019

[42] Beck M , Glössl J , Grubisic A , Spranger J . Heterogeneity of Morquio disease. Clin Genet. 1986;29(4):325‐331. 3087664 10.1111/j.1399-0004.1986.tb01262.x

[43] Nelson J , Broadhead D , Mossman J . Clinical findings in 12 patients with MPS IV a (Morquio's disease). Further evidence for heterogeneity. Part I: clinical and biochemical findings. Clin Genet. 1988;33:111‐120. 3129221 10.1111/j.1399-0004.1988.tb03421.x

[44] Bleier M , Yuskiv N , Priest T , Moisa Popurs MA . Stockler‐Ipsiroglu S Morquio‐B patient/caregiver survey: first insight into the natural course of a rare GLB1 related condition. Mol Genet Metab Rep. 2018;16:57‐63. 30094186 10.1016/j.ymgmr.2018.06.006 PMC6072644

[45] Khan S , Alméciga‐Díaz CJ , Sawamoto K , et al. Mucopolysaccharidosis IVA and glycosaminoglycans. Mol Genet Metab. 2017;120(1–2):78‐95. 27979613 10.1016/j.ymgme.2016.11.007 PMC5293636

[46] Montano A , Tomatsu S , Gottesman G , Smith M , Orii T . International Morquio A registry: clinical manifestation and natural course of Morquio A disease. J Inherit Metab Dis. 2007;30:165‐174. 17347914 10.1007/s10545-007-0529-7

[47] Northover H , Cowie RA , Wraith JE . Mucopolysaccharidosis type IVA (Morquio syndrome): a clinical review. J Inherit Metab Dis. 1996;19(3):357‐365. 8803780 10.1007/BF01799267

[48] McCarter JD , Burgoyne DL , Miao S , Zhang S , Callahan JW , Withers SG . Identification of Glu‐268 as the catalytic nucleophile of human lysosomal galactosidase precursor by mass spectrometry. J Biol Chem. 1997;272(1):396‐400. 8995274 10.1074/jbc.272.1.396

[49] Caciotti A , Donati MA , Bardelli T , et al. Primary and secondary elastin‐binding protein defect leads to impaired elastogenesis in fibroblasts from GM1‐gangliosidosis patients. Am J Pathol. 2005;167(6):1689‐1698. 16314480 10.1016/S0002-9440(10)61251-5 PMC1613190

[50] Morrone A , Bardelli T , Donati MA , et al. β‐Galactosidase gene mutations affecting the lysosomal enzyem and the elastin‐binding protein in GM1‐gangliosidosis patients with cardiac involvement. Hum Mutat. 2000;15:354‐366. 10737981 10.1002/(SICI)1098-1004(200004)15:4<354::AID-HUMU8>3.0.CO;2-L

[51] Batzios SP , Zafeiriou DI , Papakonstantinou E . Extracellular matrix components: an intricate network of possible biomarkers for lysosomal storage disorders? FEBS Lett. 2013;587(8):1258‐1267. 23454643 10.1016/j.febslet.2013.02.035

[52] Doherty C , Averill LW , Theroux M , et al. Natural history of Morquio A patient with tracheal obstruction from birth to death. Mol Genet Metab Rep. 2017;14:59‐67. 29326877 10.1016/j.ymgmr.2017.11.005 PMC5758848

[53] Yasuda E , Fushimi K , Suzuki Y , et al. Pathogenesis of Morquio A syndrome: an autopsied case reveals systemic storage disorder. Mol Genet Metab. 2013;109(3):301‐311. 23683769 10.1016/j.ymgme.2013.04.009

[54] Okumiya A , Sakuraba H , Kase R , Sugiura T . Imbalanced substrate specificity of mutant beta‐galactosidase in patients with Morquio‐B disease. Mol Genet Metab. 2003;78:51‐58. 12559848 10.1016/s1096-7192(02)00199-3

[55] Paschke E , Kresse H . Morquio disease, type B: activation of GM1‐beta‐galactosidase by GM1‐activator protein. Biochem Biophys Res Commun. 1982;109:568‐575. 6817758 10.1016/0006-291x(82)91759-4

[56] Morita M , Saito S , Ikeda K , et al. Structural bases of GM1 gangliosidosis and Morquio‐B disease. J Hum Genet. 2009;54(9):510‐515. 19644515 10.1038/jhg.2009.70

[57] Khan SA , Mason RW , Giugliani R , et al. Glycosaminoglycans analysis in blood and urine of patients with mucopolysaccharidosis. Mol Genet Metab. 2018;125(1–2):44‐52. 29779903 10.1016/j.ymgme.2018.04.011 PMC6175648

[58] Caciotti A , Donati MA , d'Azzo A , et al. The potential action of galactose as a "chemical chaperone":increase of ß‐galactosidase activity in fibroblasts from an adult GM1 gangliosidosis patient. Eur J Paediatr Neurol. 2009;13:160‐164. 18571950 10.1016/j.ejpn.2008.03.004

[59] Front S , Biela‐Banaś A , Burda P , et al. (5aR)‐5a‐C‐Pentyl‐4‐epi‐isofagomine: a powerful inhibitor of lysosomal β‐galactosidase and a remarkable chaperone for mutations associated with GM1‐gangliosidosis and Morquio disease type B. Eur J Med Chem. 2017;126:160‐170. 27750150 10.1016/j.ejmech.2016.09.095

[60] Lebl R , Thonhofer M , Tysoe C , et al. A Morita‐Baylis‐Hillman based route to C‐5a‐chain‐extended 4‐epi‐isofagomine type glycosidase inhibitors. Carbohydr Res. 2017;442:31‐40. 28288345 10.1016/j.carres.2017.03.003

[61] Rigat BA , Tropak MB , Buttner J , et al. Evaluation of of N‐nonyl‐deoxygalactonojirimycin as a pharmacological chaperone for human GM1 gangliosidosis leads to identification of a feline model suitable for testing enzyme enhancement therapy. Mol Genet Metab. 2012;107:203‐212. 22784478 10.1016/j.ymgme.2012.06.007 PMC4010500

[62] Thonhofer M , Weber P , Gonzalez Santana A , et al. Synthesis of C‐5a‐substituted derivatives of 4‐epi‐isofagomine: notable β‐galactosidase inhibitors and activity promotors of GM1‐gangliosidosis related human lysosomal b‐galactosidase mutant R201C. Carbohydr Res. 2016;429:71‐80. 27063389 10.1016/j.carres.2016.03.020

[63] Iwasaki H , Watanabe H , Iida M , et al. Fibroblast screening for chaperone therapy in γ‐galactosidosis. Brain and Development. 2006;28(8):482‐486. 16617000 10.1016/j.braindev.2006.02.002

[64] Conzelmann E , Sandhoff K . Partial enzyme deficiencies: residual activities and the development of neurological disorders. Dev Neurosci. 1983‐1984;6:58‐71. 6421563 10.1159/000112332

[65] Leinekugel P , Michel S , Conzelmann E , Sandhoff K . Quantitative correlation between the residual activity of beta‐hexosaminidase a and arylsulfatase a and the severity of the resulting lysosomal storage disease. Hum Genet. 1992;88:513‐523. 1348043 10.1007/BF00219337

[66] Deodato F , Procopio E , Rampazzo A , et al. The treatment of juvenile/adult GM1‐gangliosidosis with Miglustat may reverse disease progression. Metab Brain Dis. 2017;32(5):1529‐1536. 28577204 10.1007/s11011-017-0044-y

[67] Matsuda J , Suzuki O , Oshima A , et al. Chemical chaperone therapy for brain pathology in GM1‐gangliosidosis. Proc Natl Acad Sci. 2003;100(26):15912‐15917. 14676316 10.1073/pnas.2536657100 PMC307667

[68] Weismann CM , Ferreira J , Keeler AM , et al. Systemic AAV9 gene transfer in adult GM1 gangliosidosis mice reduces lysosomal storage in CNS and extends lifespan. Hum Mol Genet. 2015;24(15):4353‐4364. 25964428 10.1093/hmg/ddv168 PMC4492398

